# Human 2-oxoglutarate-dependent oxygenases: nutrient sensors, stress responders, and disease mediators

**DOI:** 10.1042/BST20190333

**Published:** 2020-09-28

**Authors:** Sally C. Fletcher, Mathew L. Coleman

**Affiliations:** Institute of Cancer and Genomic Sciences, University of Birmingham, Birmingham B15 2TT, U.K.

**Keywords:** disease, hydroxylation, hypoxia, nutrient sensing, oxygenase, post translational modification

## Abstract

Fe(II)/2-oxoglutarate (2OG)-dependent oxygenases are a conserved enzyme class that catalyse diverse oxidative reactions across nature. In humans, these enzymes hydroxylate a broad range of biological substrates including DNA, RNA, proteins and some metabolic intermediates. Correspondingly, members of the 2OG-dependent oxygenase superfamily have been linked to fundamental biological processes, and found dysregulated in numerous human diseases. Such findings have stimulated efforts to understand both the biochemical activities and cellular functions of these enzymes, as many have been poorly studied. In this review, we focus on human 2OG-dependent oxygenases catalysing the hydroxylation of protein and polynucleotide substrates. We discuss their modulation by changes in the cellular microenvironment, particularly with respect to oxygen, iron, 2OG and the effects of oncometabolites. We also describe emerging evidence that these enzymes are responsive to cellular stresses including hypoxia and DNA damage. Moreover, we examine how dysregulation of 2OG-dependent oxygenases is associated with human disease, and the apparent paradoxical role for some of these enzymes during cancer development. Finally, we discuss some of the challenges associated with assigning biochemical activities and cellular functions to 2OG-dependent oxygenases.

## Introduction

Fe(II)/2-oxoglutarate (2OG)-dependent oxygenases (henceforth simplified to ‘2OG-oxygenases’) catalyse a broad range of oxidative reactions across multiple biological kingdoms [1]. Diversity in reaction chemistry is particularly prominent in microorganisms where 2OG-oxygenases are reported to catalyse, amongst others, halogenation, desaturation and ring transformation reactions, in addition to hydroxylation [2]. In contrast, 2OG-oxygenase-catalysed reactions currently known in animals are limited to hydroxylation and demethylation (via hydroxylation).

Interest in protein hydroxylation in humans was fuelled by the identification of 2OG-oxygenases during studies of collagen biosynthesis, where these enzymes were found to catalyse hydroxylation of prolyl and lysyl residues. Moreover, the association between abnormal collagen hydroxylase activity and the connective tissue disorder Ehler–Danlos syndrome, which causes joint hypermobility and fragile skin, was an early indication that dysregulation of 2OG-oxygenases has repercussions for human health. [3]. Subsequently, 2OG-oxygenases have been shown to catalyse a wide range of modifications in humans, not limited just to protein substrates; hydroxylation has been demonstrated for DNA, RNA and lipids, in addition to proteins, and is now known to regulate diverse biological processes [4,5]. Accordingly, hydroxylation plays important roles across all aspects of gene expression, from epigenetic regulation through to translation control. Functional diversity of 2OG-oxygenases is mirrored by wide-ranging involvement in human disease, including cardiac and pulmonary diseases, neurological disorders, in addition to cancer [6]. Therefore, the enzymes catalysing this modification warrant further attention.

## 2OG-oxygenases

**Figure 1.**
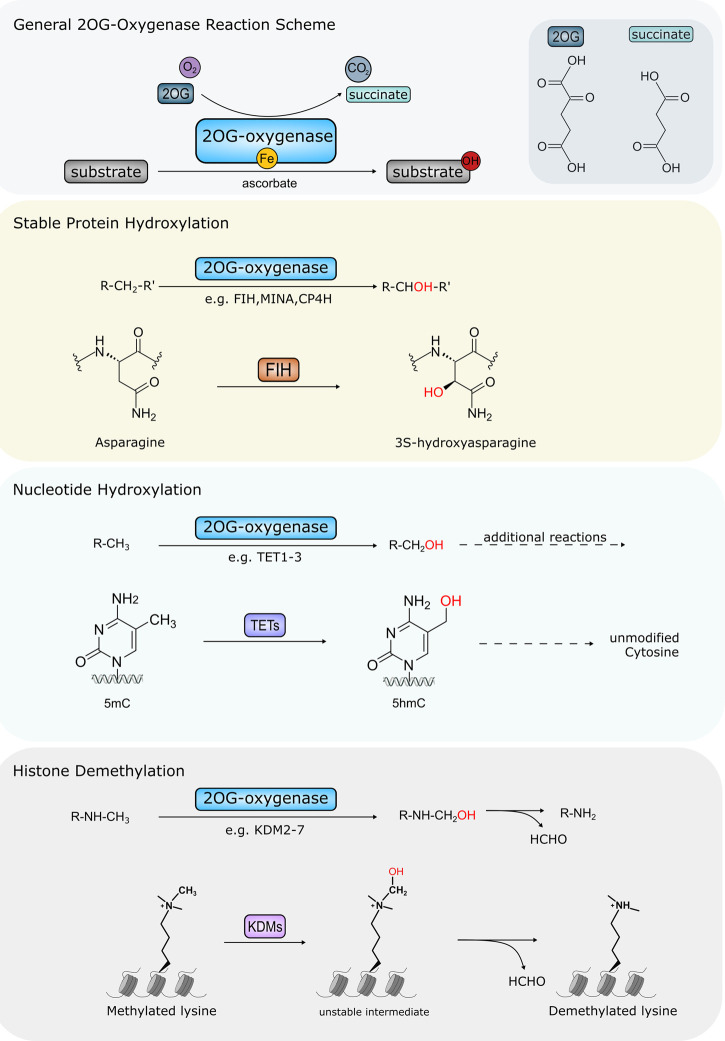
Major oxidative modifications catalysed by Human 2OG-oxygenases. Top panel: Overview of 2OG-oxygenase reaction. Substrates are hydroxylated in a reaction requiring oxygen, 2OG and iron as cofactors. Succinate and CO_2_ are released as by-products in addition to the hydroxylated substrate. Catalytic centre of 2OG-oxygenases contains bound iron. Some 2OG-oxygenases also require ascorbate for optimal activity. Chemical structures of 2OG and succinate are shown inset. Second panel: Stable protein hydroxylation. For clarity only FIH-mediated asparagine hydroxylation is shown. However, hydroxylation of arginine, aspartate, histidine, lysine and proline residues is carried out by other 2OG-oxygenases. Third panel: nucleotide hydroxylation mediated by TET enzymes. TETs hydroxylate 5mC (5-methylcytosine) at the methyl group forming 5hmC (5-hydroxymethylcytosine). This is the first stage in a multi-step oxidation to facilitate removal of 5mC modification in DNA. Bottom panel: 2OG-oxygenase-mediated histone demethylation via hydroxylation creates an unstable intermediate that spontaneously decomposes into the unmethylated form, also releasing formaldehyde (HCHO). Only demethylation of a mono-methylated lysine residue is shown. However, 2OG oxygenase-mediated demethylation also occurs at di- and tri-methylated residues. The requirement for oxygen, 2OG and iron, as well as the CO_2_ and succinate by-products are omitted for simplicity in panels 2–4: see top panel for details.

Humans have 60–70 2OG-oxygenases, so named for their requirement for Krebs cycle intermediate 2OG in addition to oxygen, Fe(II), and in some cases ascorbate (Vitamin C) [7] (Figure 1); these enzymes catalyse oxidative modifications including hydroxylation and demethylation (which initially proceeds via a hydroxylation reaction yielding an unstable intermediate). Members of the 2OG-oxygenase family share a common catalytic domain comprising a distorted double-stranded β helix (DSBH) fold made up of eight antiparallel β strands forming a barrel-like structure. (We refer the reader to [4] for further information about structural analyses of 2OG-oxygenases). The core fold brings together key amino acid side chains in the correct structural configuration necessary for coordinating cofactor and substrate binding. Fe(II) is coordinated by a conserved HxD/E…H motif whereas residues involved in 2OG, and particularly substrate binding, are more variable [8,9]. Most oxygenases also contain additional non-catalytic domains some of which aid subcellular/substrate recognition [6]. These include DNA and methylated histone binding domains for enzymes catalysing histone demethylation, for example.

The catalytic mechanism of 2OG-oxygenases has been studied extensively. It is proposed that most 2OG-oxygenases follow a conserved sequential reaction mechanism, which has been refined by experimental evidence from an initial proposal published in 1982 [4,10]. Briefly, catalysis is initiated by 2OG binding into the active site followed by the target substrate and, subsequently oxygen [8]. Oxidative decarboxylation of 2OG generates a highly reactive intermediate, which mediates hydroxylation of the target substrate releasing CO_2_ and succinate. In contrast, hydroxylation of methylated histones yields an unstable intermediate which spontaneously decomposes to produce formaldehyde and the unmethylated substrate (Figure 1). We refer the reader to other review articles [2,8] for a detailed insight into the catalytic mechanism of 2OG-oxygenases.

Of this 2OG-oxygenase superfamily ∼45 hydroxylases/demethylases modify protein substrates (including histone and non-histone targets), with the remaining oxygenases modifying other biological macromolecules. Other 2OG-oxygenase family members include the Ten-Eleven Translocation (TET) sub-family (*TET1–3*), which begin a multi-step process to remove 5-methylcytosine (5mC) modifications to DNA (Figure 1). TETs hydroxylate the methyl group in 5mC, forming 5hmC (5-hydroxymethylcytosine), which can then be further oxidised to facilitate return to an unmodified cytosine [11]. The AlkB family are also 2OG-oxygenase family members, comprising nine distinct genes in humans (*ALKBH1-8* and *FTO*). ALKBH2 and ALKBH3 are well-studied DNA repair enzymes, which remove cytotoxic methylated DNA base lesions including 1-methyladenine (1meA) and 3-methylcytosine (3meC) [12]. Other AlkB family members, including ALKBH5 and FTO instead remove RNA modifications [13,14]. 2OG-oxygenases targeting small molecules, such as those involved in fatty acid biology, are not discussed here. The reader is directed to other review articles [2,15].

**Figure 2.**
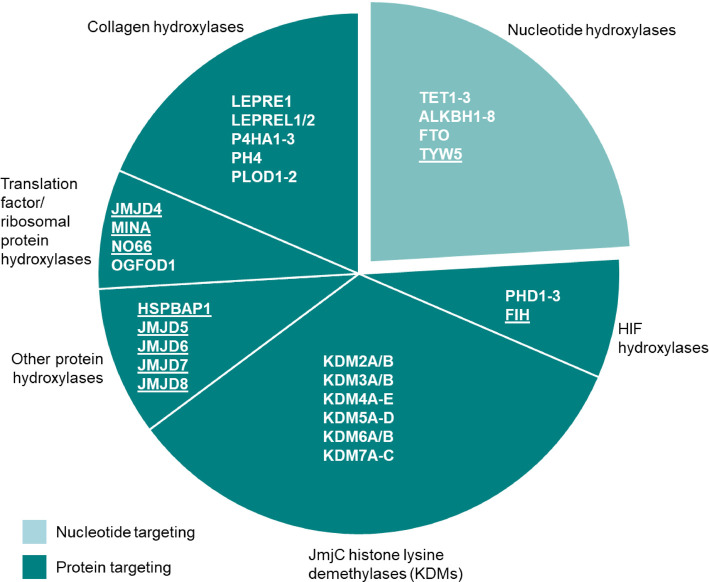
Functional grouping of 2OG-oxygenases. Nucleotide hydroxylases are shown in the light turquoise segment. These include enzymes targeting DNA (e.g. TET1–3) and RNA (e.g. TYW5). Protein-targeting enzymes are shown in the dark turquoise segments. Underlining indicates 2OG-oxygenase family members that can be phylogenetically classified as JmjC-only hydroxylases. NB: Not all members of the 2OG-oxygenase family are shown here (small molecule oxygenases have been omitted).

Protein hydroxylases/demethylases can be further subdivided according to function (Figure 2). To date, protein hydroxylases have been reported to catalyse hydroxylation of arginine, asparagine, aspartate, histidine, lysine and proline residues. Individual enzymes typically modify only a single type of amino acid in specific substrates [5]. The significant target site specificity of these enzymes is also exemplified by the precise position and stereochemistry of the resulting hydroxyl modification. For more detailed information we refer the reader to [9]. Protein hydroxylase/demethylase sub-groups include the prolyl and lysyl collagen hydroxylases as well as the hypoxia-inducible factor (HIF) hydroxylases discussed below. The largest of these sub-groups, the phylogenetically distinct KDM (lysine demethylase) family comprising ∼20 enzymes, contain a variant of the catalytic DSBH known as a Jumonji-C (JmjC) domain and catalyse histone demethylation [6,16]. The JmjC-domain, however, is not limited to catalysing histone demethylation. A further sub-group that is phylogenetically distinct to the KDMs is the JmjC-only family, which also contain this JmjC domain. Most are known, or predicted, to catalyse protein hydroxylation, as opposed to the demethylation via hydroxylation reaction catalysed by JmjC KDMs (Figure 2). However, members of this sub-family are poorly understood and represent an evolving area of research as the precise biological functions and physiological substrates of several of these JmjC-only enzymes are uncertain [17].

The aim of this review is to provide an overview of how 2OG-oxygenases interact with the cellular microenvironment, respond to cellular stresses, and how dysregulation contributes to disease propagation.

## 2OG-oxygenases: molecular sensors modified by their environment

2OG-oxygenases require fundamental nutrients as cofactors, suggesting that their activity may be altered by changes in the cellular microenvironment. In fact, such reliance has led some to propose that these enzymes may function as metabolic sensors [18,19]. Although much of the current literature is directed towards the oxygen-dependence of the HIF hydroxylases, there is widening coverage of other 2OG-oxygenases affected by cofactor availability and altered metabolism. Moreover, this is not limited just to the availability of these cofactors, but also to antagonism by oncometabolites through dysregulation of the Krebs cycle. Therefore, several questions arise about the link between pathogenesis, corresponding alterations to the cellular microenvironment, and how dysregulation of these enzymes under pathogenic conditions might contribute to disease progression.

## Oxygen

**Figure 3.**
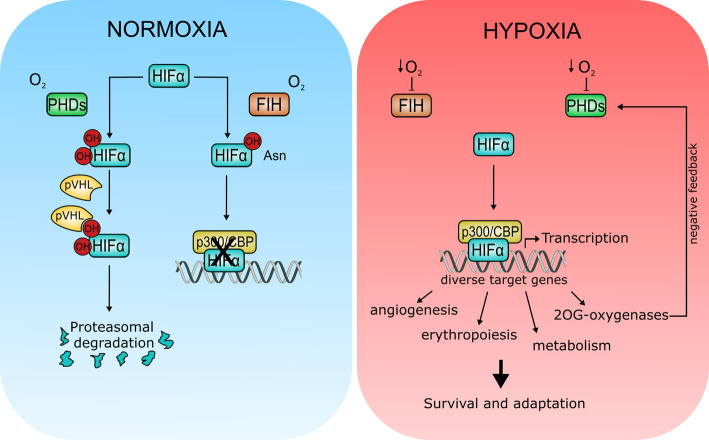
Regulation of hypoxia signalling by 2OG-oxygenases. During normoxia (left panel), prolyl hydroxylases (PHD1–3) hydroxylate conserved proline residues in the HIFα subunit creating a recognition motif for the von Hippel–Lindau (pVHL) E3 ubiquitin ligase. Hydroxylation leads to HIFα degradation via the proteasome. HIFα is also hydroxylated at a conserved asparagine residue by FIH. Hydroxylation at this site impairs HIF transactivation activity by inhibiting binding to transcriptional coactivator p300/CBP. Note that these two hydroxylation pathways are not necessarily mutually exclusive. For clarity, the requirement for 2OG and iron, as well as the CO_2_ and succinate by-products are omitted in the PHD and FIH reaction scheme (See Figure 1, Panel 1 for general reaction scheme). Under hypoxia (right panel), inhibition of PHDs and FIH leads to HIFα stabilisation and activation. HIFα activation results in transcription of genes involved in a wide range of cellular processes to promote survival and adaptation under low oxygen conditions. Some 2OG-oxygenases are also HIFα transcriptional targets, including PHD2/3 which form part of a negative feedback loop to limit HIF activity.

As 2OG-oxygenases require oxygen for catalysis, it is reasonable to suggest that their enzymatic activity may be sensitive to fluctuations in cellular oxygen levels. Moreover, variability in oxygen tensions across different tissues, and the frequency of hypoxic regions in solid tumours highlights the importance for cells to sense and adapt to changes in oxygen availability. In fact, the mechanism behind oxygen sensing was recognised in the 2019 Nobel Prize in Physiology or Medicine, uncovering a key role for 2OG-oxygenases in this fundamental cellular process through regulation of HIF (Figure 3).

HIF is an oxygen-dependent transcription factor, which, during anoxic or low oxygen conditions induces the expression of genes regulating survival, angiogenesis and invasion, amongst others [20]. In the presence of oxygen, HIF protein is degraded thus preventing inappropriate activation of these genes. Three 2OG-oxygenases containing a prolyl hydroxylase domain (PHD1–3 or EGLN1–3) hydroxylate two conserved prolyl residues in the HIFα subunit. These modifications create a recognition motif for the von Hippel–Lindau (pVHL) E3 ubiquitin ligase, resulting in HIF ubiquitination and subsequent degradation via the proteasome [21]. Hydroxylation-mediated regulation of HIF activity also occurs via another 2OG-oxygenase, FIH (Factor Inhibiting HIF). In this case, HIF transactivation activity at normal oxygen levels (normoxia) is impaired through FIH-dependent hydroxylation of an asparagine residue in one of the C-terminal transactivation domains (Figure 3, left panel). HIF hydroxylation at this residue reduces HIF transcriptional activity by inhibiting binding to the transcriptional coactivator p300/CBP [22]. The catalytic activity of HIF hydroxylases is reduced under low oxygen levels [23,24]. Therefore, reduced oxygen availability during hypoxia causes inhibition of these enzymes, and the subsequent stabilisation and activation of HIF. HIF activation leads to induction of HIF target genes, including some 2OG-oxygenases, to promote survival and adaptation (Figure 3, right panel).

Importantly, PHD1–3 are suited to function as oxygen sensors as they have relatively low affinity for oxygen and are therefore able to sense oxygen fluctuations in a physiologically relevant range [18]. These findings have raised interest in whether other 2OG-oxygenases may also behave as oxygen sensors. Previous *in vitro* experiments suggest that oxygen affinity varies between 2OG-oxygenases indicating differential sensitivity across the wider family. For example, protein hydroxylases JMJD4, MINA, NO66 and OGFOD1, which are proposed to have cellular roles linked to protein translation [25], have been shown to retain significant catalytic activity under severe hypoxia (0.1% O_2_) [26–28]. Histone methylation is increased during hypoxia, although the exact mechanism is not understood [29–31]. One hypothesis suggests increased histone methylation is due to modified lysine demethylase activity under low oxygen conditions. In fact, recent studies have shown that KDM4A, KDM5A, and KDM6A are oxygen-sensitive [32–34]. Similarly, TET enzyme activity is reduced at pathophysiological levels of oxygen present in solid tumours, leading to hypermethylation at gene promoters [35]. Although the TET enzymes are unlikely to be physiological oxygen sensors [36], hypermethylation of tumour suppressor genes in hypoxia, as a consequence of TET enzyme inhibition, provides a compelling example for the negative consequences of inhibiting hydroxylation during tumourigenesis, and therefore the important defensive role performed by some 2OG-oxygenases in disease prevention.

There is also some evidence that oxygen binding in the active site can be competitively inhibited by nitric oxide. In fact, nitric oxide has been shown capable of inhibiting PHD1–3 as well as a number of histone demethylases, although the biological role of these findings is unknown [37–39].

Beyond the HIF hydroxylases, the extent to which other 2OG-oxygenases are oxygen-sensitive, however, is not really well-characterised but warrants further investigation, especially with a view to understanding how disease progression during hypoxia may be affected by changes in hydroxylation activity. For example, genomic instability is a well-known hallmark of cancer intimately associated with hypoxia [40–42], and there is a growing appreciation of the role of 2OG-oxygenases in genome biology and the response to DNA damage (discussed in more detail below). A complete understanding of the oxygen sensitivities of 2OG-oxygenases will therefore help determine whether their potential inhibition in low oxygen may contribute to tumourigenesis.

## Iron

Iron is an essential cofactor for 2OG-oxygenase activity (reviewed in [4]), meaning that these enzymes may be sensitive to fluctuations in iron homeostasis. As such, iron chelators such as desferrioxamine have been shown both *in vitro* and *in vivo* to inhibit HIF hydroxylases most likely through the removal of bound iron in the enzyme active site [43]. Moreover, 2OG-oxygenases may also be sensitive to the presence of heavy metal ions in cells. Divalent transition metal ions, including manganese, nickel, cobalt, copper and zinc have all been shown to inhibit 2OG-oxygenases, through displacement of iron from the catalytic pocket [44]. In particular, sensitivity to nickel inhibition has been demonstrated for enzymes across the 2OG-superfamily including HIF hydroxylase PHD2, lysine demethylase KDM3A, and DNA repair enzyme ALKBH3 [45] suggesting that some 2OG-oxygenases may be sensors of transition metals.

## Ascorbate

The need for ascorbate in 2OG-oxygenase catalysis has been known since the realisation that scurvy symptoms are linked to ascorbate-deficiency, and defective collagen formation. Hydroxyproline stabilises collagen helices; its formation is catalysed by a 2OG-oxygenase, collagen prolyl 4-hydroxylase (CP4H), in a reaction requiring ascorbate [46]. Ascorbate deficiency, therefore, limits hydroxyproline availability leading to reduced collagen stability and potential deterioration of connective tissues. It is proposed that ascorbate helps maintain iron in the active site in the reduced Fe^2+^ state required for catalytic activity (reviewed in [47]). But how widely this applies across the 2OG family remains unclear, as not all 2OG-oxygenases require ascorbate for activity [7].

There is still interest in understanding the role that ascorbate plays in regulating these enzymes. For example, there is some suggestion that the HIF hydroxylases, especially FIH, may be sensitive to perturbations in ascorbate levels [48]. HIF transcriptional activity, and to a lesser extent protein stabilisation induced by iron competition, could be inhibited by ascorbate treatment in some cancer cell lines [49]. In addition, ascorbate may be important in epigenetic regulation, through both histone and DNA demethylation. For example, by inducing activity of histone demethylases KDM2A/B, ascorbate has been shown to promote reprogramming of induced pluripotent stem cells [50]. Similarly, ascorbate treatment of mouse embryonic stem cells led to a global reduction in H3K9me2 methylation mediated by KDM3A/B [51]. Moreover, multiple studies [52–55] have demonstrated that ascorbate can stimulate TET enzyme activity. However, further research is clearly required to appreciate the role of ascorbate in 2OG-oxygenase biology.

## 2OG

2OG is a central intermediate at the crossroads of key metabolic pathways. It is produced from isocitrate, for example, by isocitrate dehydrogenases (IDH1–3). 2OG is also generated from the catabolism of amino acids, including glutamine. Intracellular 2OG concentrations can therefore be affected by multiple factors, and variations are known to occur in biological processes such as ageing [56]. Therefore, understanding how sensitive different 2OG-oxygenases are to 2OG availability deserves attention. Glutamine catabolism, via glutamate, is a major cellular source of 2OG, particularly in cancer cells. It has been shown that glutamine-deficiency can lead to DNA damage accumulation due to inhibition of ALKBH enzymes as a result of 2OG-depletion [57]. Moreover, increasing cellular 2OG levels also affects some 2OG-oxygenases. Embryonic stem cells given 2OG show increased self-renewal capacity, through a mechanism linked to KDM6 and TET enzyme activity [58]. Moreover, cell-permeating 2OG was shown to overcome PHD inhibition in cancer cell models [59]. Interestingly, loss of PHD2 has been shown to increase cellular 2OG levels, which the authors suggest is due to PHD2 being a major ‘sink’ for 2OG [60]. This raises the intriguing possibility that 2OG availability, and its impact on the activity of some 2OG-oxygenases, could be affected by the abundance and activity of other members of the enzyme family.

Together, the studies described above suggest that 2OG-oxygenases are sensitive to fluctuations in cellular 2OG concentrations, although further research is needed to understand how such oscillations may affect the activity of other family members. Moreover, the HIF hydroxylases have been proposed as metabolic sensors. In addition to oxygen sensing, it is hypothesised that they may indirectly sense changes in amino acid levels through 2OG availability, as amino acid starvation has been shown to inhibit the PHDs through 2OG depletion [19]. Whether other 2OG-oxygenases may similarly behave as nutrient sensors deserves further attention.

## Oncometabolites

Dysregulated metabolism, promoting sustained cell growth and proliferation, is now recognised as a fundamental step during tumourigenesis. Mutations in the genes encoding Krebs cycle enzymes fumarate hydratase (*FH*) and succinate dehydrogenase (*SDH*), amongst others, are associated with cancer development. Moreover, the gain of function mutations in specific isoforms of isocitrate dehydrogenase (*IDH1 and 2*), are also linked to cancer [61,62]. Mutations in *FH, SDH* and *IDH1/2* are associated with carcinogenesis through an abnormal accumulation of metabolic intermediates or ‘oncometabolites’, including fumarate, succinate or D-2-hydroxyglutarate (D-2HG), respectively. Mutations in *IDH1* and *IDH2* typically cause a single amino acid substitution in the active site that confers a neomorphic enzymatic activity resulting in the conversion of 2OG to antagonist D-2HG [63]. *IDH* mutations are found in a wide variety of cancer types including gliomas, cartilaginous tumours and acute myeloid leukaemia (AML) [64]. Inactivating mutations in *SDH* and *FH* have been associated with cancers including paraganglioma/pheochromocytomas (PPGL) and hereditary leiomyomatosis and renal cell cancer (HLRCC), amongst others [62,65], and lead to elevated levels of succinate and fumarate, respectively. We refer the reader to recent reviews [66–68] for detailed discussions about the biology of oncometabolites.

**Figure 4.**
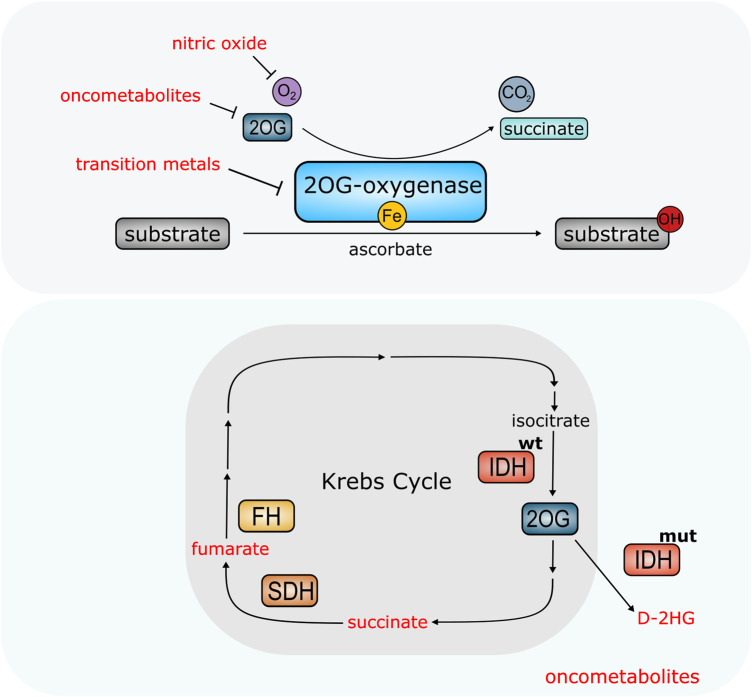
2OG-oxygenases are molecular sensors modified by the cellular environment. Top panel: Availability of nutrients (ascorbate, iron, oxygen) and metabolite 2OG affect activity of 2OG-oxygenases. Other molecules can also interfere with oxygenase activity. Nitric oxide can compete with oxygen. Certain transition metals can also displace iron from the catalytic site, blocking enzymatic activity. High levels of oncometabolites can compete with 2OG and inhibit 2OG-oxygenase activity in cancer. Bottom panel: Elevated levels of oncometabolites succinate and fumarate result from mutations in Succinate Dehydrogenase (SH) and Fumarate Hydratase (FH) which form part of the Krebs Cycle. Mutations in Isocitrate Dehydrogenase (IDH^mut^) confer neomorphic enzymatic activity resulting in the conversion of 2OG into oncometabolite D-2HG.

Oncometabolites share structural similarity with 2OG, which fuelled interest in understanding how 2OG-oxygenase biology might be implicated in tumourigenesis. Studies have shown that D-2HG, succinate, and fumarate can competitively inhibit multiple 2OG-oxygenases including the TET proteins, ALKBH enzymes, PHDs and a number of histone demethylases from the KDM family (Figure 4) [36,69–74]. Moreover, the L-enantiomer of 2HG (L-2HG) has also been shown to inhibit the PHDs and TET enzymes [75]. However, the evidence thus far indicates that not all 2OG-oxygenases behave equally. For example, the TET enzymes are inhibited more strongly by fumarate and succinate *in vitro* than D-2HG [36]. Therefore, a major challenge in the 2OG-oxygenase field is to understand which enzymes are inhibited by each oncometabolite*,* whether altered activity is transferrable to behaviour in tumours, and whether inhibition is actually causative in cancer.

Pseudohypoxia, the abnormal stabilisation of HIF proteins in the absence of hypoxia, is a clear example of such an area for further study. Pseudohypoxia is observed in cancers with *SDH* and *FH* mutations and is proposed to occur due to succinate/fumarate-induced inhibition of the HIF hydroxylases [76]. Because hypoxia is known to promote pro-tumourigenic processes such as metastasis, correspondingly, pseudohypoxia has been postulated as a causative agent in such oncometabolite-driven tumours. However, whether pseudohypoxia is really a driver remains unclear, as HIF inhibition in different studies reported opposing outcomes [77,78]. Moreover, evidence suggests that *VHL*-mutated tumours show a stronger activation of HIF target genes compared with tumours with *SDH* mutations [68]. Therefore, it is likely that other mechanisms may contribute to oncometabolite-driven tumourigenesis, potentially through inhibition of other 2OG-oxygenases. For example, the elevated levels of both fumarate and succinate in HLRCC and PPGL, have been shown to suppress homologous recombination through KDM4A/B inhibition [79]. Moreover, Morin and colleagues recently reported that pseudohypoxia and TET inhibition may act synergistically in *SDH*-mutated tumours [80].

Several lines of evidence indicate that 2OG-oxygenase inhibition by D-2HG suppresses DNA repair. For example, ALKBH inhibition sensitises *IDH-*mutant cells to alkylating agents [72]. Furthermore, cell lines expressing altered IDH1 showed increased endogenous formation of DNA double-strand breaks (DSBs) linked to KDM4A/B inhibition [81]. Finally, D-2HG has been implicated in the down-regulation of DNA damage response regulator ATM in *IDH1-*mutated AML, through increased H3K9 methylation [82]. These findings suggest that elevated D-2HG levels in cancer may exacerbate tumourigenesis through increased mutation rates from inhibition of DNA repair, potentially through repression of 2OG-oxygenases. Moreover, since some members of the 2OG-oxygenase family remain poorly characterised, the full landscape of oncometabolite targets is unknown, raising the possibility that inhibition of other, currently understudied, 2OG-oxygenases may also contribute to tumourigenesis.

Overall, there is compelling evidence that these enzymes can be regulated by changes in the intracellular environment (Figure 4), although further research particularly *in vivo* is needed to better understand this. In particular, careful consideration should be given to the distinction between a bona fide *sensor,* that might be responsible for translating an environmental change into a physiological response, versus an enzyme that is simply *sensitive* to cofactor abundance. Several 2OG-oxygenases are clearly sensitive to cofactor availability, but clear criteria are needed to help define whether some family members might behave as nutrient sensors, akin to the role of prolyl hydroxylases in oxygen sensing.

In addition to regulation by microenvironmental changes, increasing evidence suggests that 2OG-oxygenases may be directly targeted in cellular stress responses.

## 2OG-oxygenases: Stress responders

Cells encounter multiple types of stress. DNA damage, for example, risks the integrity of the genome. Perturbations to cellular homeostasis are also problematic if cells are unable to adapt accordingly. Increasing evidence suggests that 2OG-oxygenases may perform important roles in helping cells respond appropriately to a variety of stresses.

Hypoxia is one example of such a stress. Multiple 2OG-oxygenases, including several KDMs, are HIF1α targets, and are therefore up-regulated during hypoxia (reviewed in [43]). For example, KDM3A is induced in hypoxia, which regulates transcription of pro-survival genes such as *HMOX1* during hypoxic adaptation [83]. The HIF hydroxylases PHD2 and PHD3, are also HIF1α transcriptional targets, and are thought to function as part of a negative feedback loop to precisely control HIF activity, including upon reoxygenation [21]. Other 2OG-oxygenases, such as ALKBH5, which carries out N6-methyladenosine (m6A)-demethylation of RNA [84], are also induced in a HIF1α-dependent manner in hypoxia. However, the function of this activity is uncertain [85]. Whether hypoxia-inducibility has evolved to maintain a critical activity threshold of specific oxygen-sensitive 2OG-oxygenases, or simply reflects an important biological role of these enzymes in the adaptive response to hypoxia, is not yet clear.

**Figure 5.**
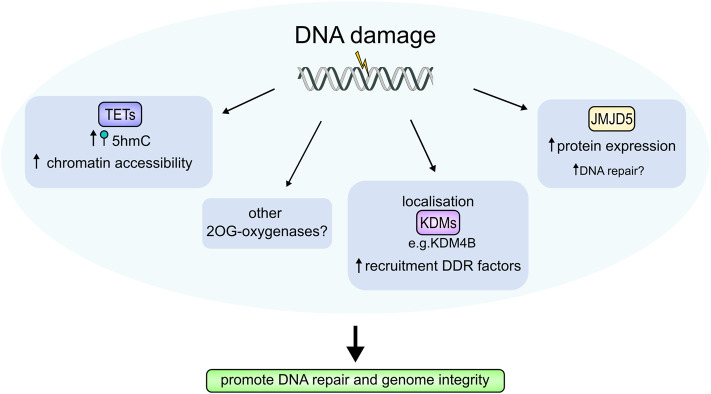
2OG-oxygenases in the DNA damage response (DDR). 2OG-oxygenases contribute to the cellular response to DNA damage through several mechanisms. Elevated 5hmC levels, produced by the TET enzymes are found at DNA damage sites and are thought to increase chromatin accessibility to repair complexes. Multiple KDMs localise to sites of DNA damage, which enhances recruitment of DDR proteins. JMJD5 protein levels are also increased by DNA damage, although its precise function in the DDR is unknown. Damage-dependent regulation of 2OG-oxygenases is thought to facilitate DNA repair thereby promoting genome integrity. Other 2OG-oxygenases may also be involved.

Hypoxia is not the only cell stress to which 2OG-oxygenases are responsive. Indeed, multiple 2OG-oxygenases are thought to regulate DNA repair (Figure 5). For example, histone demethylases KDM2A, KDM4B, KDM4D, and KDM5B are recruited to sites of DNA damage to facilitate DNA repair [86–89], through both histone demethylation-dependent and -independent mechanisms. 5hmC, produced by TET1–3, accumulates at sites of endogenous DNA damage suggesting the TET enzymes also promote genome integrity through the DNA damage response (DDR); the authors propose 5hmC accumulation may improve chromatin accessibility for repair proteins [90]. Moreover, JMJD5, an arginyl hydroxylase in the JmjC-only subfamily [91], has been linked to homologous recombination and mismatch repair [92,93]. JMJD5 is also induced in response to DNA damaging agents [94], suggesting it is important in the DDR. Another member of the JmjC-only family, lysyl hydroxylase JMJD6, has also recently been proposed to play a role in the DDR [95,96].

Dynamic regulation of protein translation control is needed to help cells adapt to environmental stresses. Prolyl hydroxylase OGFOD1, which modifies translational accuracy through ribosomal hydroxylation, has been linked to this process [28,97,98]. Loss of OGFOD1 in multiple cell models has been associated with stress granule formation, a marker of translational stress [28,99]. These findings suggest OGFOD1 is important for preventing translational stress by promoting proper ribosome function.

The above examples demonstrate that 2OG-oxygenases are involved in fundamental cellular processes, and the adaptive responses needed to promote organism survival. It is therefore not surprising that aberrant function of these enzymes is associated with the disease.

## 2OG-oxygenases: disease mediators

Stress-responsive proteins that mediate adaptive responses (e.g. p53), often play critical roles in cellular homeostasis, and are therefore commonly implicated in disease. A number of different disorders have been associated with members of the 2OG-oxygenase family, reflecting the wide range of biological functions regulated by these enzymes and their substrates [5,6]. These disorders include cardiac and pulmonary diseases, neurological disorders, obesity as well as cancer. In fact, multiple 2OG-oxygenases have been associated with neurological disorders. For example, mutations in the KDM6 sub-family have been linked with intellectual disability [100,101]. Other KDMs, including KDM4C, KDM5C and KDM7B have been associated with autism spectrum disorders, through a SNP, missense mutation, and in-frame deletion, respectively [6]. Similarly, mutations in JMJD7, a JmjC-only lysyl hydroxylase catalysing hydroxylation of TRAFAC GTPases [102] have also been found in patients with intellectual disabilities and autism [103,104].

Several studies have linked JmjC-only histidyl hydroxylase MINA with a role in the inflammatory response. MINA, which hydroxylates a conserved histidine on large ribosomal subunit component Rpl27a [27], was identified as an important factor regulating differentiation of pro-inflammatory T cells [105]. In addition, a SNP in the MINA gene has been linked with an increased risk of asthma [106]. Furthermore, *Mina*-deficient mice showed decreased airway inflammation in response to allergens [107].

For many disease-associated 2OG-oxygenases, the mechanisms involved are often not fully understood, particularly with respect to the links between pathophysiological findings and the biological substrates of the corresponding enzyme. These uncertainties are especially relevant in cancer where association studies have ascribed both pro- and anti-tumourigenic roles to different 2OG-oxygenases.

Many studies have investigated how 2OG-oxygenases behave in cancer. The reader is referred to [5,11,17,108] for more detailed reviews. For some enzymes, for example, TET2 and the KDM4 sub-family, their role is relatively clear-cut. KDM4A–D are over-expressed in a wide variety of tumour types, and are therefore suggested to play a pro-tumourigenic role in cancer (reviewed in [109]). In contrast, TET2 is thought to be a tumour suppressor, and is either mutated or dysregulated in multiple cancers, particularly haematological malignancies. In fact, TET2 is one of the most commonly mutated genes in AML [110]. For other 2OG-oxygenases however, particularly those in the JmjC-only sub-family, the picture is less clear [17,111]. In the case of arginyl hydroxylase JMJD5, for example, multiple studies have described both pro- and anti-tumourigenic functions. In support of a pro-oncogenic role in cancer, high JMJD5 expression has been correlated with increased metastasis, invasiveness and poor survival in breast, colorectal, prostate, and oral cancers [112–115]. Conversely, a multi-cohort retrospective study across ten cancer types has suggested that JMJD5 may function as a tumour suppressor; high expression levels were associated with a lower risk of death in pancreatic and liver cancer cohorts, while low JMJD5 levels correlated with poorer survival outcomes [116]. In fact, JMJD5 was originally identified in a tumour suppressor gene screen [93]. Down-regulation of JMJD5 in hepatocellular carcinoma, cholangiocarcinoma and lung cancer further supports the tumour suppressor assignment [117–119]. Taken together, the conflicting assignments suggest there is likely a context-dependence to the role of these enzymes in cancer development. Moreover, given that both under- and over-expression have been linked with malignancy, it is clear that both the level and enzymatic activity of these proteins must be finely tuned to guard against disease.

## Inhibition

Given that 2OG-oxygenases have been implicated in major diseases, it is unsurprising that they have attracted attention as potential therapeutic targets, particularly as the active sites are amenable to small-molecule inhibition [44]. In fact, inhibitors of HIF prolyl hydroxylases have been in clinical trials for treating renal anaemia, with interest in whether PHD inhibition may also be beneficial for managing ischaemia [120]. Therapeutic KDM4 inhibitors are also of interest, given the proposed role for the KDM4 sub-family in promoting tumour growth. However, the development of potent and selective inhibitors has been challenging [109]. Stringent specificity for such inhibitors is paramount, particularly as 2OG-oxygenases have wide-ranging cellular roles. Moreover, elucidating the true enzymatic function and biological substrates of some 2OG-oxygenases will be crucial to their success as clinical targets.

## Activity assignment controversy

One of the major challenges in the hydroxylation field is resolving controversies over the biochemical assignment of some 2OG-oxygenases, particularly in the JmjC-only sub-family [17]. For example, different studies have attributed histone demethylase and histone tail clipping activity to JMJD5 [94,112,121], whilst detailed biochemical and structural studies indicate that it functions as a protein hydroxylase [91,122]. A similar investigation into published JMJD6 substrates was also unable to confirm other proposed targets and biochemical activities beyond lysyl hydroxylation [123], including a reported histone arginyl demethylase activity [124]. In fact, initial assignments of histone demethylase activity (or declaration of KDM activity even in the absence of evidence), is a theme among the JmjC-only sub-family of 2OG-oxygenases (as discussed in [16,17,111]) (e.g. MINA, NO66, JMJD4, and JMJD7). Although the reasons are unclear, it could perhaps relate to some confusion over the biochemical potential of the JmjC domain (i.e. that it is not limited to KDM activity). In fact, detailed evolutionary and structural analyses indicate that the JmjC domain of KDMs likely evolved from a prokaryotic JmjC-only protein hydroxylase [125].

Unfortunately, controversy is not limited to biochemical activity assignments, but also extends to the specific biological targets of those activities: A comprehensive investigation into the 20+ reported non-HIF substrates of HIF prolyl hydroxylases did not confirm any hydroxylation activity against the reported novel targets [126]. These findings have been discussed in detail by others [127,128]. Important factors contributing to the challenges of identifying hydroxylase substrates likely include complexities related to detection and quantification. Thus far the only reliable methodology for these is mass spectrometry (MS), which has inherent limitations with respect to site localisation, particularly regarding novel substrate residues and artefactual oxidations. Overall, the studies discussed above highlight the need for very careful consideration of putative substrate assignments for other 2OG-oxygenases, including combinatorial approaches based on quantitative MS and detailed biochemical and structural analyses. Despite the uncertainties surrounding the biological targets of some of these enzymes, it is clear from disease models that 2OG-oxygenases play important roles in multiple cellular contexts. Therefore, further studies that accurately define the biochemical activities and targets of 2OG-oxygenases, particularly ‘orphan’ enzymes, is warranted.

## Conclusion

Hydroxylation is an evolving area of research. 2OG-oxygenases are a broad enzyme family that perform important roles in regulating key physiological processes. Reliance on key metabolites for activity means that these enzymes are sensitive to changes in the cellular microenvironment, potentially enabling them to act as nutrient sensors (Figure 4). Functional roles are seemingly not just limited to sensing cellular stresses, however, but also responding to them, helping cells adapt to changes in the environment (Figure 5). The importance of these enzymes for survival is also further highlighted by their dysregulation in disease. However, unanswered questions about enzymes in this family still remain. In particular, within the JmjC-only sub-family, further research is required to understand the biological significance of hydroxylation catalysed by these enzymes, and to unravel the paradoxical roles ascribed to these proteins in complex diseases such as cancer.

## Perspectives

- **Importance of the field:** Human 2OG-oxygenases are a broad enzyme family that catalyse hydroxylation, regulating fundamental biological processes. These enzymes are increasingly associated with wide-ranging human diseases including cancer, which has fuelled interest in developing biochemically specific and clinically relevant inhibitors.
- **Summary of current thinking:** Reliance on key nutrients such as oxygen for activity means that 2OG-oxygenases are sensitive to changes in the cellular microenvironment and may act as nutrient sensors. Emerging evidence also indicates that 2OG-oxygenases are important for responding to cellular stresses including hypoxia and DNA damage to help cells adapt to changes in the environment.
- **Future directions:** Many unanswered questions remain in the 2OG-oxygenase field, particularly with respect to accurately defining the biochemical activities and targets of human 2OG-oxygenases, especially ‘orphan' enzymes. Such studies are particularly required in the JmjC-only sub-family where further research is necessary to understand the biological significance of hydroxylation catalysed by these enzymes.

## Open Access

Open access for this article was enabled by the participation of University of Birmingham in an all-inclusive *Read & Publish* pilot with Portland Press and the Biochemical Society under a transformative agreement with JISC.

## Abbreviations

2OG: 2-oxoglutarate
AML: acute myeloid leukaemia
D-2HG: D-2-hydroxyglutarate
DDR: DNA damage response
DSBH: double-stranded β helix
FH: Fumarate Hydratase
FIH: Factor Inhibiting HIF
HIF: hypoxia-inducible factor
HLRCC: hereditary leiomyomatosis and renal cell cancer
IDH: Isocitrate Dehydrogenase
KDM: Lysine Demethylase
MS: mass spectrometry
PPGL: paraganglioma/pheochromocytomas
SDH: Succinate Dehydrogenase
TET: Ten-Eleven Translocation
